# Mesenchymal stem cells alleviate experimental immune-mediated liver injury via chitinase 3-like protein 1-mediated T cell suppression

**DOI:** 10.1038/s41419-021-03524-y

**Published:** 2021-03-04

**Authors:** Qiuli Liu, Xiaoyong Chen, Chang Liu, Lijie Pan, Xinmei Kang, Yanli Li, Cong Du, Shuai Dong, Andy Peng Xiang, Yan Xu, Qi Zhang

**Affiliations:** 1 Biotherapy Center, The Third Affiliated Hospital of Sun Yat-sen University, 510630 Guangzhou, China; 2 Key Laboratory for Stem Cells and Tissue Engineering, Center for Stem Cell Biology and Tissue Engineering, Ministry of Education, Sun Yat-sen University, 510080 Guangzhou, China; 3 Department of Pathophysiology, Zhongshan School of Medicine, Sun Yat-sen University, 510080 Guangzhou, China; 4 Cell-gene Therapy Translational Medicine Research Center, The Third Affiliated Hospital of Sun Yat-sen University, 510630 Guangzhou, China; 5 Guangdong Provincial Key Laboratory of Liver Disease Research, The Third Affiliated Hospital, Sun Yat-sen University, 510630 Guangzhou, China

**Keywords:** T cells, Mesenchymal stem cells, Autoimmune hepatitis

## Abstract

Liver diseases with different pathogenesis share common pathways of immune-mediated injury. Chitinase-3-like protein 1 (CHI3L1) was induced in both acute and chronic liver injuries, and recent studies reported that it possesses an immunosuppressive ability. CHI3L1 was also expressed in mesenchymal stem cells (MSCs), thus we investigates the role of CHI3L1 in MSC-based therapy for immune-mediated liver injury here. We found that CHI3L1 was highly expressed in human umbilical cord MSCs (hUC-MSCs). Downregulating CHI3L1 mitigated the ability of hUC-MSCs to inhibit T cell activation, proliferation and inflammatory cytokine secretion in vitro. Using Concanavalin A (Con A)-induced liver injury mouse model, we found that silencing CHI3L1 significantly abrogated the hUC-MSCs-mediated alleviation of liver injury, accompanying by weakened suppressive effects on infiltration and activation of hepatic T cells, and secretion of pro-inflammatory cytokines. In addition, recombinant CHI3L1 (rCHI3L1) administration inhibited the proliferation and function of activated T cells, and alleviated the Con A-induced liver injury in mice. Mechanistically, gene set enrichment analysis showed that JAK/STAT signalling pathway was one of the most significantly enriched gene pathways in T cells co-cultured with hUC-MSCs with CHI3L1 knockdown, and further study revealed that CHI3L1 secreted by hUC-MSCs inhibited the STAT1/3 signalling in T cells by upregulating peroxisome proliferator-activated receptor δ (PPARδ). Collectively, our data showed that CHI3L1 was a novel MSC-secreted immunosuppressive factor and provided new insights into therapeutic treatment of immune-mediated liver injury.

## Introduction

Abnormal immune responses and immune cell infiltration, elicited by various liver injuries (such as viral or parasite infection, drug toxicity, alcohol abuse and metabolic diseases), can destroy the immune privileged state of the liver and result in liver inflammation^1^. Mesenchymal stem cells (MSCs), a multipotent progenitor cells that could differentiate into osteoblasts, adipocytes, chondrocytes, have attracted widespread attention as their unique immunomodulatory effects towards a large number of effector immune cells, including T lymphocytes, B cells, natural killer cells, dendritic cells, macrophages and neutrophils^2,3^. Preclinical and clinical studies have shown that MSC transplantation may reduce liver inflammation, thus improving regeneration of hepatocytes and serving as a promising strategy for patients with immune-mediated liver injuries^4,5^. However, the underlying mechanisms by which MSCs alleviate immune-mediated liver injuries remain not yet been fully elucidated.

Chitinase-3-like protein 1 (CHI3L1), a highly evolutionarily conserved secreted protein, have an important role in the pathogenesis of inflammatory diseases because of its upregulation in inflamed tissues of ulcerative colitis, Crohn’s disease, rheumatoid arthritis, osteoarthritis and liver cirrhosis, as well as in solid cancers^6–9^. Elevated CHI3L1 expression plays a major role in microenvironment remodelling in different diseases^10^. Recent studies revealed that CHI3L1 had an immunosuppressive function, such as, CHI3L1 negatively regulates T cell activation, and deficiency of CHI3L1 accelerates stroke development through enhancement of neuroinflammation via decreasing M2 macrophage polarisation^11–13^.

Interestingly, CHI3L1 is also reported to express on MSCs, and was increased when stimulated with cytokines or during differentiation^14,15^. In this study, using Con A-induced hepatitis, a commonly used experimental model to study immune-mediated liver injury, we identified that CHI3L1 was a key mediator of immunomodulatory function of hUC-MSCs. CHI3L1 was abundantly secreted by hUC-MSCs and knocking down CHI3L1 in hUC-MSCs alleviated its therapeutic effect on Con A-induced liver injury in mouse models. Specifically, decreasing CHI3L1 expression attenuated the ability of hUC-MSCs to inhibit CD3^+^ T cell activation and proliferation and suppress the production of tumour necrosis factor-α (TNF-α) and interferon-γ (IFN-γ) in vitro and in vivo. In contrast, administration of recombinant CHI3L1 (rCHI3L1) was sufficient to inhibit T cell proliferation and function in vitro and alleviate Con A-induced liver injury in vivo. Mechanistically, the immunosuppressive effect of hUC-MSC-derived CHI3L1 was dependent on the upregulation of PPARδ and subsequent inhibition of STAT1/3 phosphorylation in T cells. In summary, we demonstrated that CHI3L1 is a novel secreted factor that mediates the immunosuppressive effects of hUC-MSCs and provided new insights into promising therapeutics for refractory liver diseases.

## Results

### CHI3L1 was highly expressed in hUC-MSCs

MSCs have been isolated from various tissues, including bone marrow, adipose tissue, umbilical cord, and many others. Although the cells shared similar cell surface markers, MSC heterogeneity exist at multiple levels, including among tissues^16,17^. To determine the differences between MSCs isolated from different tissues, we performed RNA sequencing on MSCs isolated from human bone marrow (BM-MSCs), olfactory mucosa (OM-MSCs), and umbilical cord (UC-MSCs) (2 donors for each). The data showed that although the cells were isolated from two individuals, cells from the same tissues shared more similarities (Supplementary Fig. 1A). Interestingly, CHI3L1, an evolutionarily conserved secreted glycoprotein, was highly expressed in hUC-MSCs (Supplementary Fig. 1B). The abundant expression of CHI3L1 in hUC-MSCs compared to human BM-MSCs and OM-MSCs was validated by RT-qPCR, western blot and ELISA (Supplementary Fig. 1C-E). Since the effect of CHI3L1 in regulating immune responses, we were very interested in what role it would play in MSC-mediated immune modulation.

### MSCs inhibited the activation and proliferation of T cells through CHI3L1 in vitro

To evaluate the role of CHI3L1 in MSCs, two specific shRNAs were designed to downregulate CHI3L1 in hUC-MSCs. Effective knockdown was confirmed by RT-qPCR, western blot analysis of cell lysates, and ELISA analysis of culture supernatant (Supplementary Fig. 1F-H). Knocking down CHI3L1 did not influence the morphology, proliferation, surface marker expression (including CD90, CD105, CD73, CD45 and CD34), as well as osteogenic and adipogenic differentiation potential of hUC-MSCs (Supplementary Fig. 1I and 1J).

**Fig. 1:**
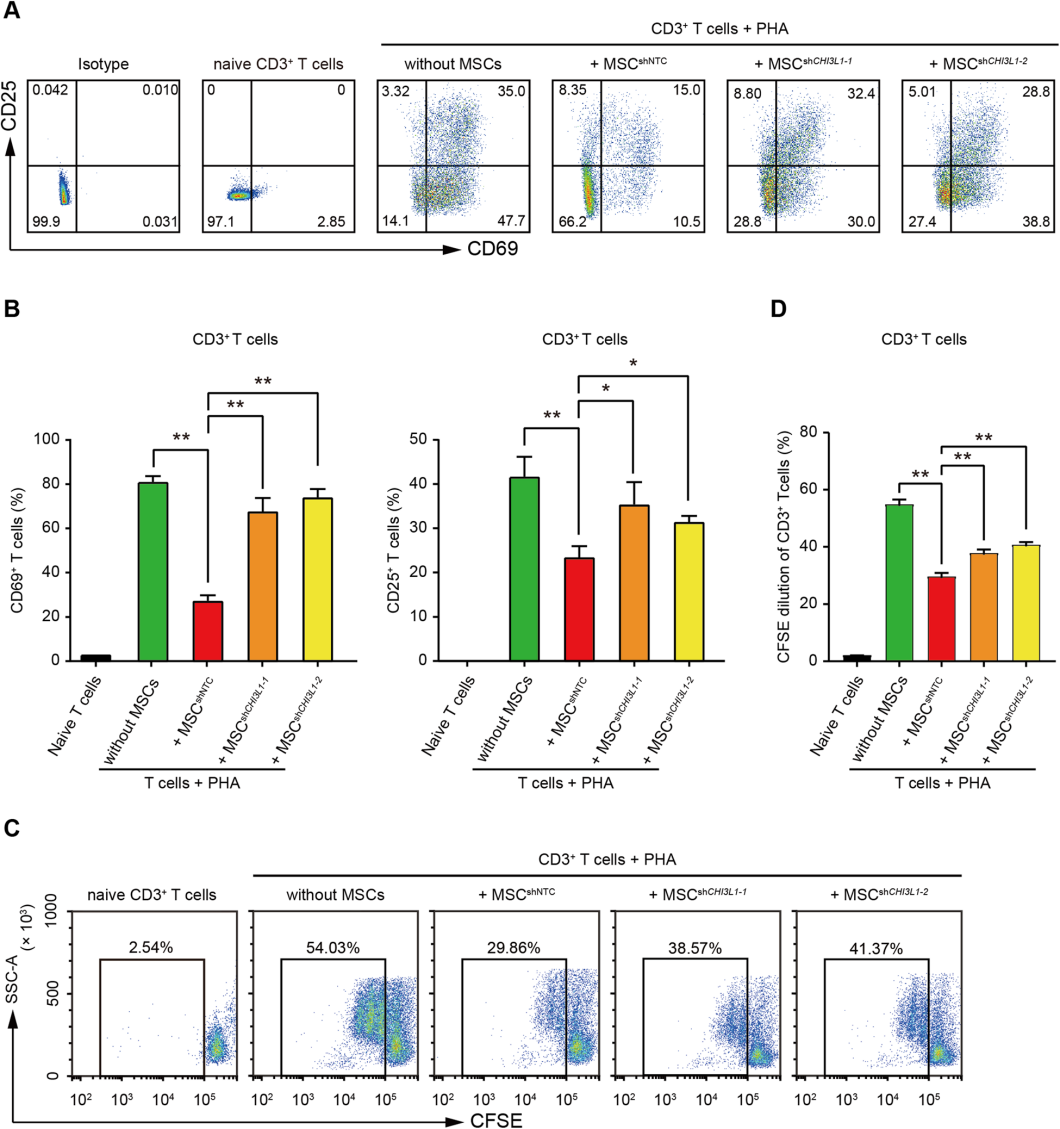
hUC-MSCs inhibit the activation and proliferation of T cells through CHI3L1 in vitro. **A** Representative flow cytometry plots of CD69 and CD25 expression on CD3^+^ T cells in indicated groups. CD3^+^CD25^-^ T cells were sorted by FACS and cultured alone (naïve T cells) or activated with PHA (2.5 μg/mL) for 24 h. For co-culture, sorted T cells were co-cultured with MSCs in the presence of PHA for 24 h. Without MSCs, activated T cells cultured alone; + MSC^shNTC^, activated T cells co-cultured with hUC-MSCs transduced with shNTC; + MSC^sh*CHI3L1*^, activated T cells co-cultured with hUC-MSCs transduced with shRNA for *CHI3L1*. **B** Quantification of CD69 (left) and CD25 (right) positive CD3^+^ T cells in (**A**). **C** Representative flow cytometry plots and quantification (**D**) of flow cytometry analysis for proliferation of CD3^+^ T cells labelled with CFSE in indicated groups. Data in (**B**) and (**D**) are shown as mean ± SD (*n* = 3 biological replicates) with the indicated significance (*p < 0.05, **p < 0.01).

Followed, we investigated the role of CHI3L1 in MSC-mediated immunomodulation using T cells/MSCs co-culture model, a classical assay for evaluating the immunosuppression of MSCs. we co-cultured human CD3^+^ T cells with hUC-MSCs and performed T cell activation and proliferation assays. CD3^+^CD25^-^ T cells were sorted by FACS and cultured alone (naïve T cells) or activated with PHA (2.5 μg/mL) for 24 h. For co-culture, sorted T cells were co-cultured with MSCs in the presence of PHA for 24 h. As shown in Figs. 1A and 1B, control MSCs (MSC^shNTC^) significantly inhibited the expression of CD69 and CD25 (two widely used surface markers of T cell activation) on CD3^+^ T cells, while the inhibitory effect was alleviated upon knockdown of CHI3L1 in hUC-MSCs (MSC^*shCHI3L1*^). Similarly, the proliferation ratio of CD3^+^ T cells was significantly inhibited by MSC^shNTC^, and downregulating CHI3L1 potently attenuated the inhibitory effect of MSCs (Fig. 1C and 1D).

**Fig. 2:**
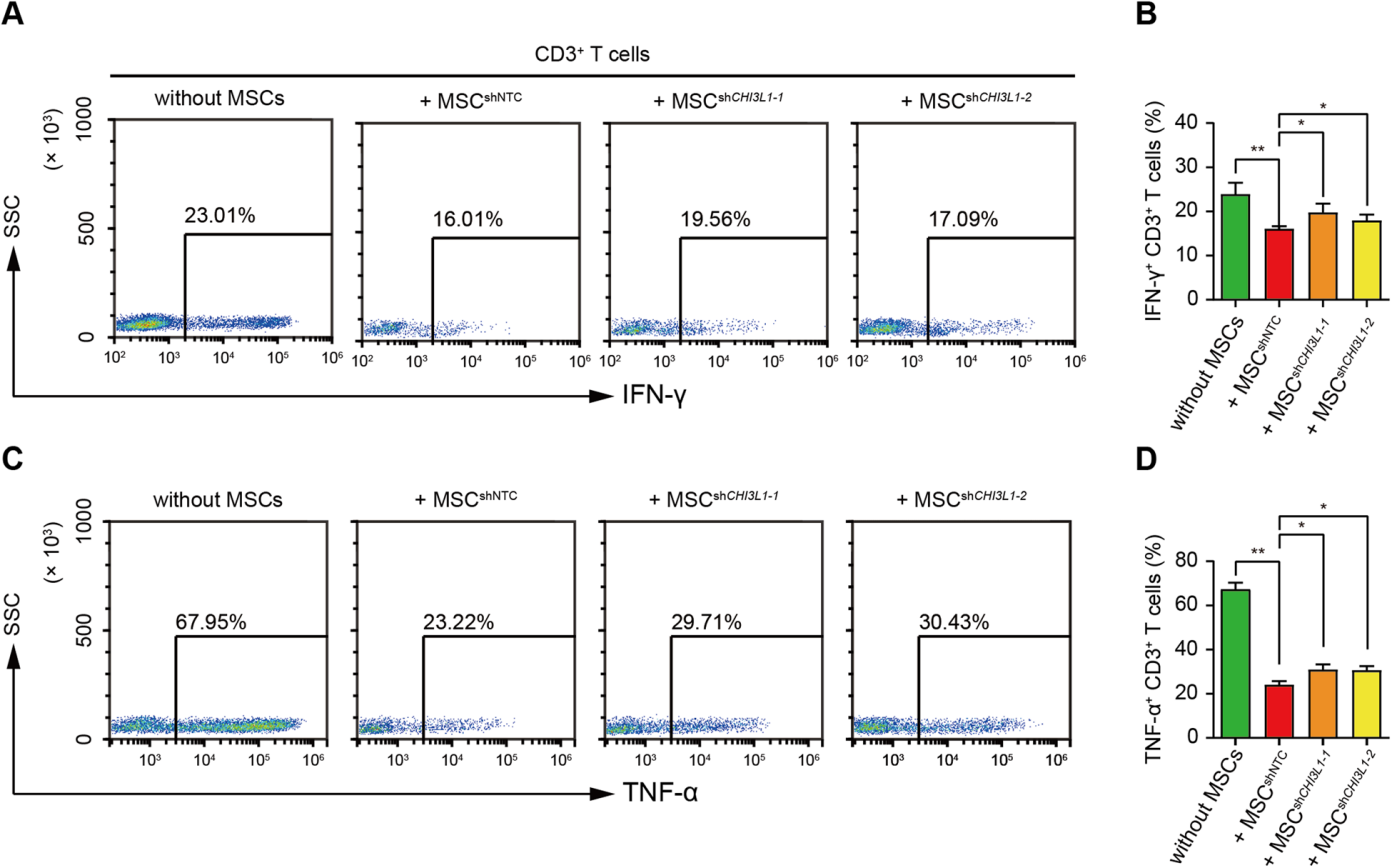
hUC-MSCs downregulate the pro-inflammatory cytokine production of CD3^+^ T cells partially through CHI3L1 in vitro. **A** Representative plots and quantification (**B**) of flow cytometry analysis for IFN-γ-producing CD3^+^ T cells in indicated groups. **C** Representative plots and quantification (**D**) of flow cytometry analysis for TNF-α-producing CD3^+^ T cells in indicated groups. Flow cytometry analysis were conducted 3 days after CD3^+^ T cells co-cultured with MSCs. Data in (**B**) and (**D**) are shown as mean ± SD (*n* = 3 biological replicates) with the indicated significance (*p < 0.05, **p < 0.01).

Activated T cells (especially CD4^+^ Th cells and CD8^+^ cytotoxic T lymphocytes (CTLs)) can classically produce a series of pro-inflammatory cytokines, including IFN-γ and TNF-α, to promote inflammation^18^. Next, we co-cultured hUC-MSCs with CD3^+^ T cells and analysed intracellular IFN-γ and TNF-α expression using FACS. MSC^shNTC^ suppressed IFN-γ and TNF-α expression in activated CD3^+^ T cells, and knocking down CHI3L1 alleviated this suppression (Fig. 2A-D). These results demonstrated that hUC-MSCs suppressed the activation, proliferation, and pro-inflammatory cytokine production of CD3^+^ T cells in vitro, and CHI3L1 was involved in the MSC-mediated T cell suppression.

### MSCs alleviated Con A-induced liver injury through CHI3L1

**Fig. 3:**
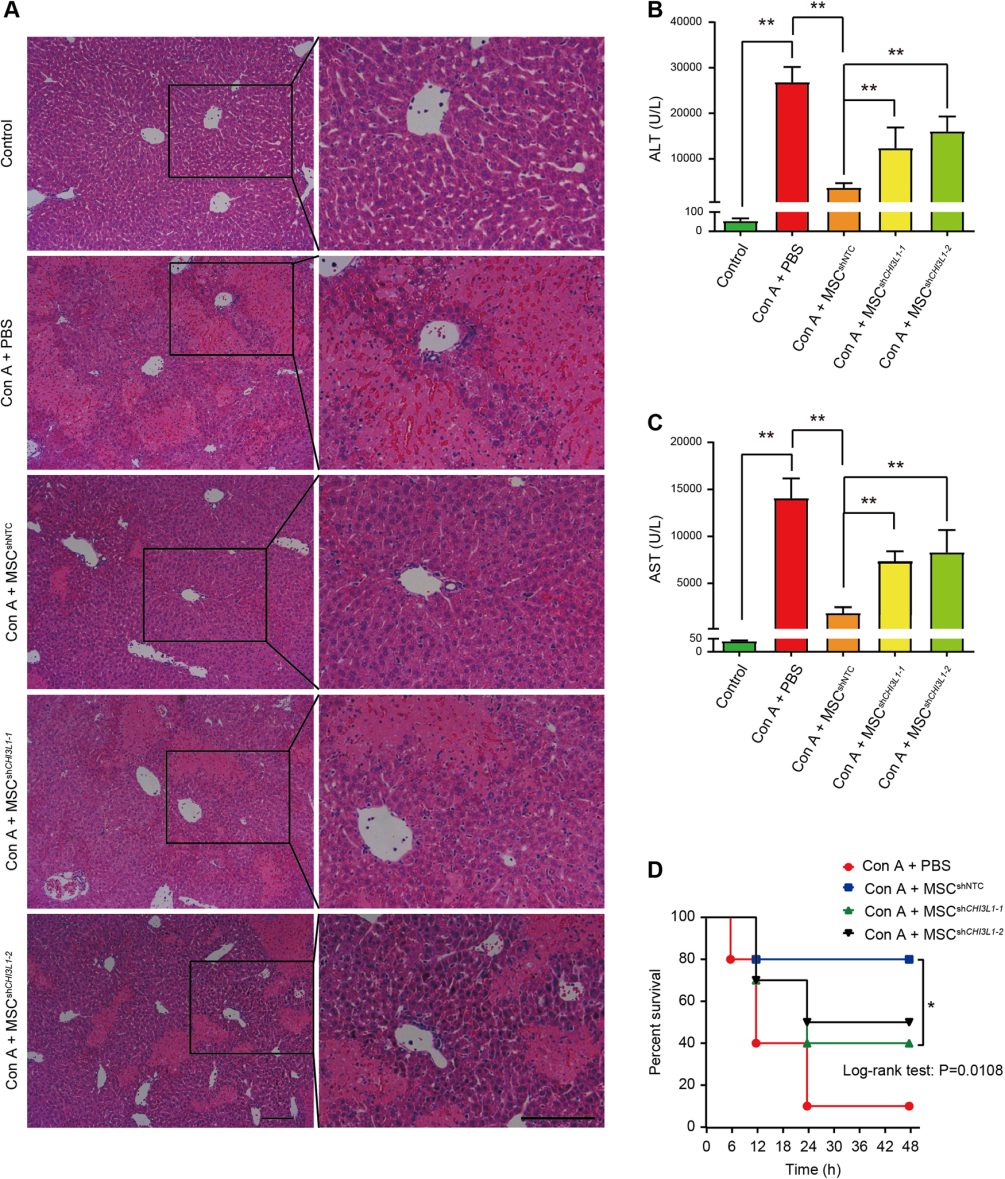
hUC-MSCs alleviate Con A-induced mouse liver injury through CHI3L1. **A** Representative Hematoxylin and eosin (H&E) staining photographs of liver tissues 24 h after Con A administration in indicated groups. Scale bar = 100 μm. **B** Serum ALT and AST (**C**) levels were measured 24 h after Con A treatment in indicated groups. **D** The survival curves of mice in indicated groups (data were collected every 6 h, *n* = 10 for each group). Data in (**B**) and (**C**) are shown as mean ± SD (*n* = 4–7 for each group) with indicated significance (*p < 0.05, **p < 0.01).

To evaluate whether CHI3L1 mediates the immunosuppressive effects of hUC-MSCs in vivo, experimental AIH, a commonly used experimental model to study immune-mediated liver injury^19^, was induced by Con A (15 mg/kg) in mice, and PBS, MSC^shNTC^ or MSC^sh*CHI3L1*^ was administered intravenously. Histological analysis after 24 h showed that, similar with MSCs from other origins^20,21^, hUC-MSCs effectively ameliorated Con A-induced hepatocyte necrosis and disseminated haemorrhage (Fig. 3A). In addition, compared to the serum from the PBS group, serum collected from the MSC^shNTC^ group showed significantly decreased levels of ALT and AST activity (two liver injury markers), indicating effective improvement of Con A-induced liver injury by hUC-MSCs. Knocking down CHI3L1 (the MSC^sh*CHI3L1*^ group) markedly abolished the therapeutic effects of hUC-MSCs, as evidenced by both histological and serum analyses (Fig. 3A-C). We also investigated the effect of hUC-MSCs on Con A-induced mortality. As expected, MSC^shNTC^ treatment improved the survival rate from 10% to 80% at 48 h, while knocking down CHI3L1 resulted in a survival rate of 40%–50% (Fig. 3D). These results showed that CHI3L1 effectively mediated the therapeutic effect of hUC-MSCs on Con A-induced hepatitis in vivo.

### MSCs suppressed T cell activation and production of pro-inflammatory cytokines in Con A-induced mouse hepatitis models through CHI3L1

**Fig. 4:**
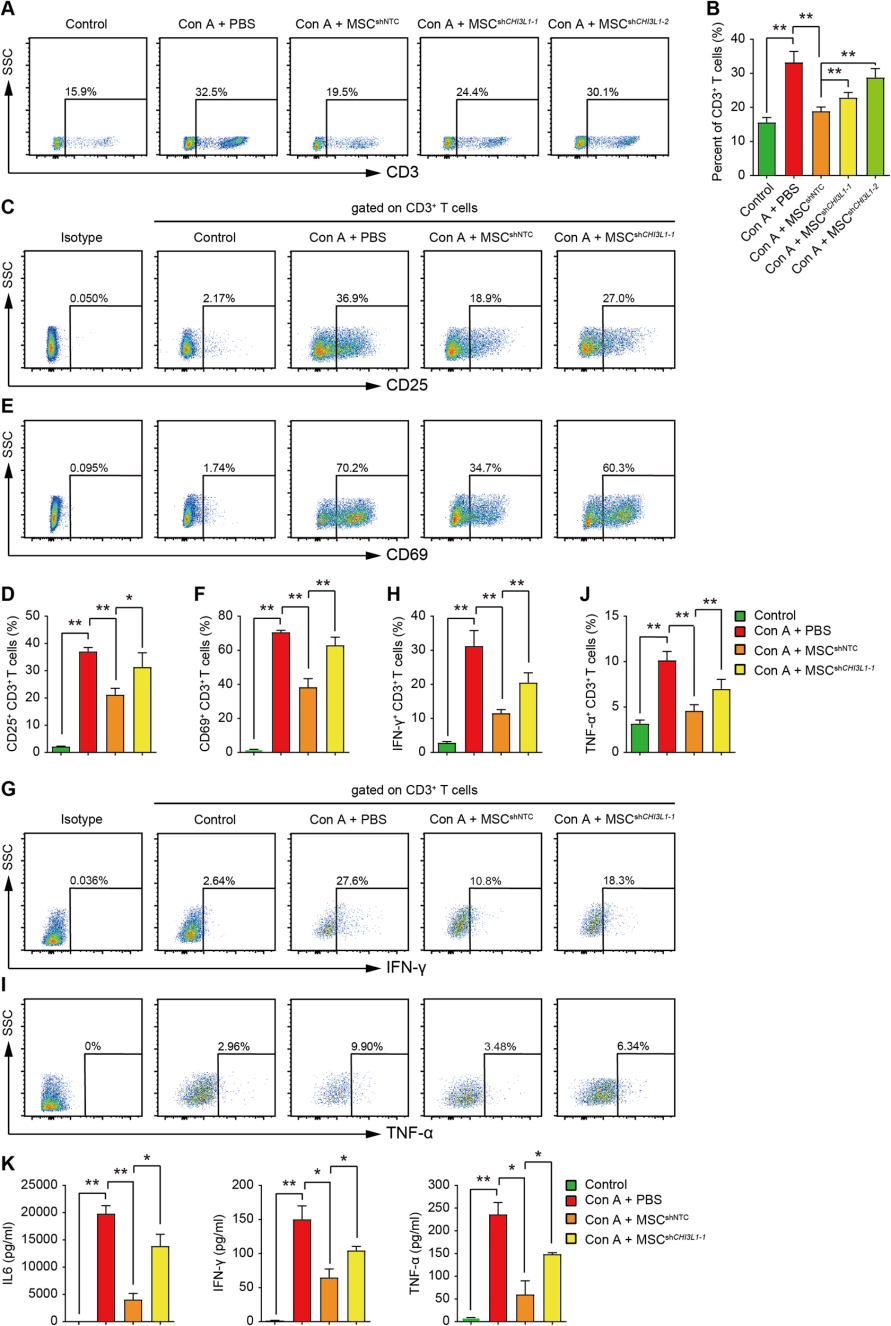
hUC-MSCs suppress the activation and pro-inflammatory cytokine secretion of hepatic T cells through CHI3L1 in Con A-induced hepatitis. **A** Representative plots and quantification (**B**) of flow cytometry analysis for CD3^+^ T cells in liver MNCs 24 h after Con A administration in indicated groups. **C**–**F** Representative plots and quantification of flow cytometry analysis for CD25 (**C**–**D**) and CD69 (**E**–**F**) expression in CD3^+^ T cells in liver MNCs 24 h after Con A administration in indicated groups. **G**–**J** Representative plots and quantification of flow cytometry analysis for IFN-γ (**G**–**H**) and TNF-α (**I**–**J**) -producing CD3^+^ T cells in liver MNCs 24 h after Con A administration in indicated groups. **K** Serum concentrations of IL6, IFN-γ, and TNF-α 24 h after Con A administration in indicated groups were detected by CBA kit. Analyses in (**G**–**J**) were conducted with liver MNCs stimulated with PMA (50 ng/mL), ionomycin (500 ng/mL) and BFA (10 μg/mL) for 6 h after isolation. Data in (**B**), (**D**), (**F**), (**H**), (**J**), and (**K**) are shown as mean ± SD (*n* = 5 for each group) with indicated significance (*p < 0.05, **p < 0.01).

Activation of immune cells (T cells, natural killer T cells, neutrophils, and macrophages) and their cytokine products, especially IFN-γ and TNF-α, has been reported to contribute to Con A-induced hepatitis^18^. MSCs could ameliorate Con A-induced hepatitis by reducing both the infiltration and activity of autoreactive T cells in the liver^20,22^. Next, we investigated whether CHI3L1 produced by hUC-MSCs could affect the number and activity of hepatic T cells in vivo. Flow cytometry analysis of CD3^+^ T cells in liver lymphocytes revealed that the number of CD3^+^ T lymphocytes was markedly reduced after MSC^shNTC^ treatment, while MSC^sh*CHI3L1*^ induced a much weaker effect in limiting CD3^+^ T lymphocyte infiltration than MSC^shNTC^ (Fig. 4A and 4B). Interestingly, analysis of hepatic T cells also showed a significant decrease in the frequency of CD69^+^ or CD25^+^ T cells in the MSC^shNTC^ group compared to the PBS group and a marked recovery of CD69^+^ and CD25^+^ T cell frequency after knocking down CHI3L1 in hUC-MSCs (Fig. 4C-F). These results indicated that CHI3L1 mediated the suppression of both infiltration and activation of hepatic CD3^+^ T cells by hUC-MSCs in Con A-induced hepatitis.

Furthermore, we performed intracellular staining to analyse the role of CHI3L1 in mediating hUC-MSC suppression on pro-inflammatory cytokine production by hepatic CD3^+^ T cells in vivo. The results showed that IFN-γ and TNF-α secretion by hepatic CD3^+^ T cells was suppressed after MSC^shNTC^ treatment compared to the PBS group. The suppression by hUC-MSCs was significantly alleviated after knocking down CHI3L1 (Fig. 4G-J). Mouse serum CBA analysis of IL6, IFN-γ, and TNF-α was consistent with the results of the intracellular cytokine staining analysis (Fig. 4K).

### Recombinant CHI3L1 protected against Con A-induced hepatic injury

**Fig. 5:**
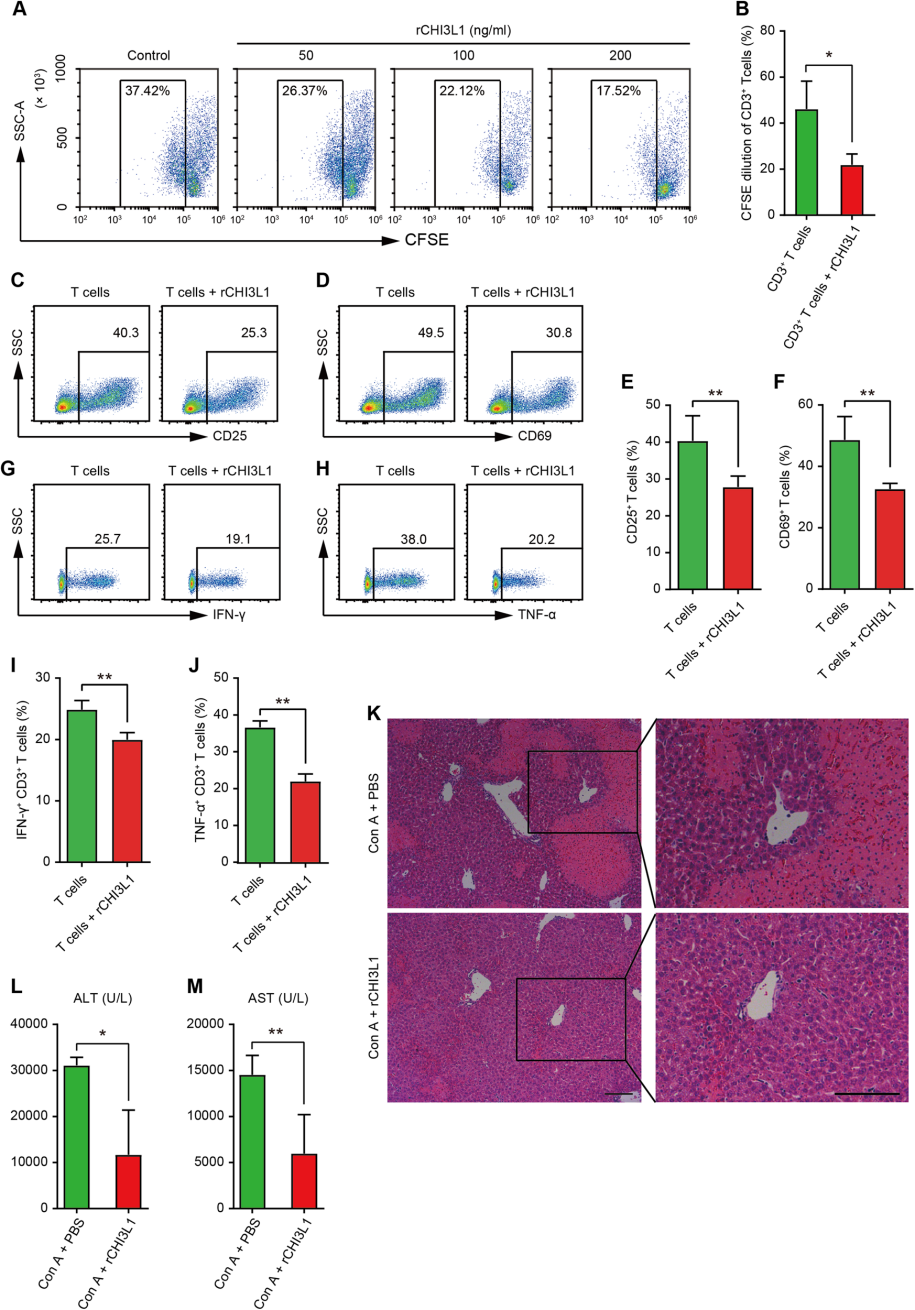
Recombinant CHI3L1 (rCHI3L1) ameliorates Con A-induced hepatitis. **A** Representative flow cytometry plots for analysing proliferation of CD3^+^ T cells labelled with CFSE treated with rCHI3L1 in indicated conditions. PBS was used as a control. **B** Quantification of flow cytometry analysis for proliferation of CD3^+^ T cells labelled with CFSE in PBS (control) or rCHI3L1 (100 ng/mL) treated group (*n* = 3 biological replicates). **C**–**F** Representative plots (**C**–**D**) and quantification (**E**–**F**) of flow cytometry analysis for CD25 and CD69 expression in CD3^+^ T cells treated with control (PBS) or rCHI3L1 (100 ng/mL) (*n* = 3 biological replicates). **G**–**J** Representative plots (**G**–**H**) and quantification (**I**–**J**) of flow cytometry analysis for IFN-γ and TNF-α expression in CD3^+^ T cells treated with control (PBS) or rCHI3L1 (100 ng/mL) (*n* = 3 biological replicates). **K** Representative H&E staining photographs of liver tissues 24 h after Con A administration in control (PBS) and rCHI3L1 (500 ng) treated groups. Scale bar = 100 μm. **L** Serum ALT and AST (**M**) levels 24 h after Con A treatment in indicated groups (*n* = 4–7 mice per group). Data in (**B**), (**E**), (**F**), (**I**), (**J**), (**L**), and (**M**) are shown as mean ± SD with indicated significance (*p < 0.05, **p < 0.01).

To further verify that CHI3L1 mediates the protective effect of hUC-MSCs on Con A-induced, T cell-mediated hepatic injury, we examined whether rCHI3L1 could inhibit the function of T cells in vitro and alleviate hepatic pathology induced by Con A in vivo. As shown in Fig. 5A and 5B, rCHI3L1 inhibited human CD3^+^ T cell proliferation in vitro in a dose-dependent manner. Next, we tested T cells activation (using CD25 and CD69 as activation markers) and pro-inflammatory cytokine production of T cells treated with rCHI3L1. rCHI3L1 significantly inhibit T cell activation (Fig. 5C-F) and secretion of IFN-γ and TNF-α (Fig. 5G-J). Consistent with the in vitro studies, administration of rCHI3L1 after Con A treatment also significantly improved liver histology and decreased the activities of serum ALT and AST (Fig. 5K-M), indicating that rCHI3L1 protected against Con A-induced liver injury.

### CHI3L1 alleviated Con A-induced hepatic injury through PPARδ/STAT1/3 signalling

**Fig. 6:**
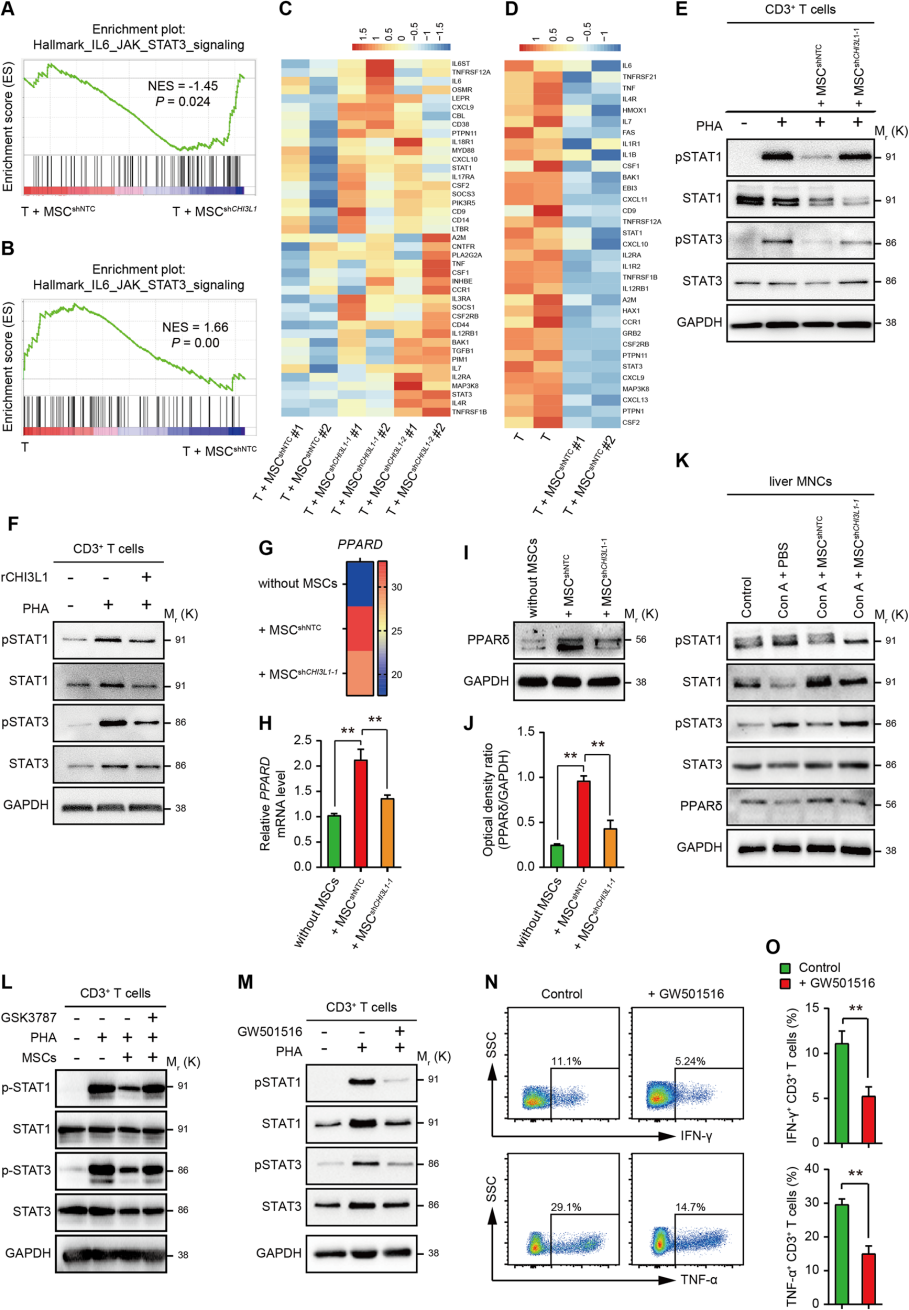
hUC-MSC-derived CHI3L1 inhibits T cells through PPARδ/STAT1/3. **A** GSEA showing that the IL6-JAK-STAT3 pathway is enriched in T cells co-cultured with MSC^sh*CHI3L1*^ compared with T + MSC^shNTC^ group. **B** GSEA showing that the IL6-JAK-STAT3 pathway is repressed in T cells co-cultured with MSC^shNTC^ compared to activated T cells. **C**–**D** Heatmap of RNA-sequencing data showing the expression level of genes involved in IL6-JAK-STAT3 pathway in indicated samples. **E** Western blot for phosphorylated and total STAT1 and STAT3 in T cells with indicated treatments for 2 days. **F** Western blot for phosphorylated and total STAT1 and STAT3 in T cells cultured with or without rCHI3L1 (100 ng/mL). **G** Heatmap of RNA-sequencing data showing the expression level of *PPARD* in activated T cells (T cells), T cells co-cultured with MSC^shNTC^ (+ MSC^shNTC^), and T cells co-cultured with MSC^sh*CHI3L1*^ (+ MSC ^sh*CHI3L1*^). **H** RT-qPCR for *PPARD* in T cells cultured alone or co-cultured with hUC-MSCs with or without CHI3L1 knockdown. **I** Western blot and corresponding densitometry analysis (**J**) for PPARδ in T cells culture alone, or co-cultured with MSC^shNTC^ or MSC^sh*CHI3L1*^. **K** Western blot for phosphorylated and total STAT1 and STAT3 in liver MNCs in indicated groups. **L** Representative western blot for testing phosphorylated-STAT1 (p-STAT1), total STAT1, phosphorylated-STAT3 (p-STAT3), and total STAT3 in human naïve CD3^+^ T cells, CD3^+^ T cells activated by PHA, activated CD3^+^ T cells co-cultured with MSCs, and activated CD3^+^ T cells co-cultured with MSCs in the presence of PPARδ inhibitor (GSK3787, S8025, Selleck). GSK3787 was added simultaneously with PHA at a concentration of 1 μM for 48 h. **M** Western blot for phosphorylated and total STAT1 and STAT3 in T cells stimulated with PHA in the absence or presence of PPARδ agonist GW501516 (10 μM). **N** Representative flow cytometry plots and quantification (**O**) of IFN-γ (upper) and TNF-α (lower)-producing CD3^+^ T cells treated with GW501516. DMSO was used as a control in (**L**–**O**).

**Fig. 7:**
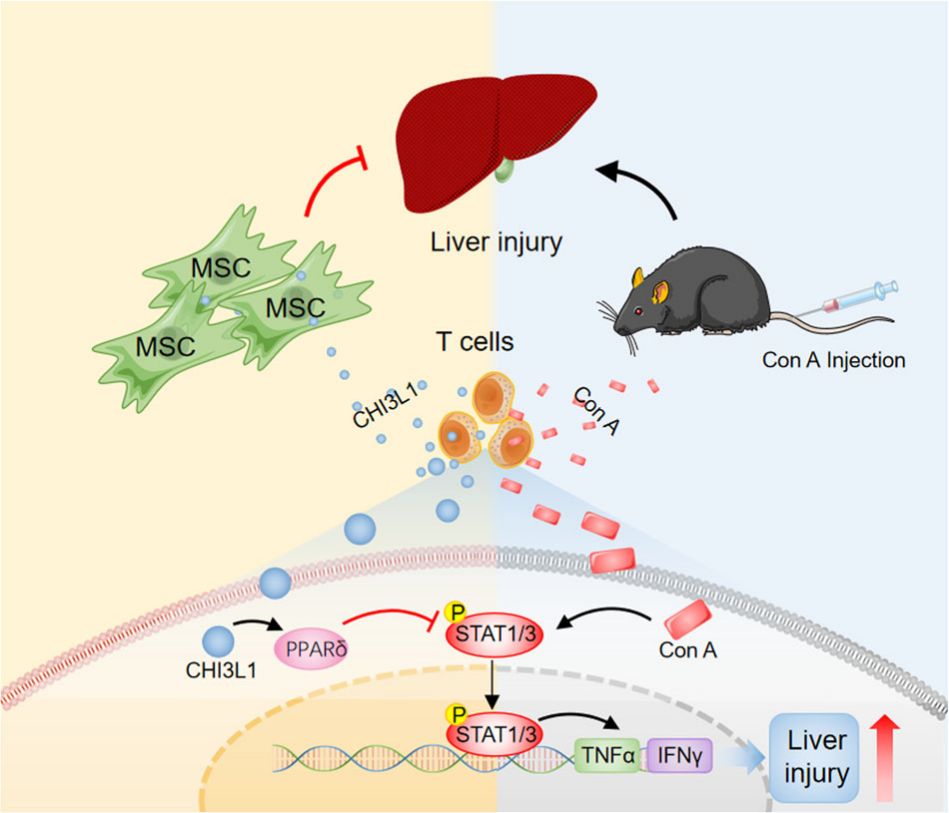
hUC-MSCs alleviate experimental immune-mediated liver injury via CHI3L1-mediated T cell suppression. Schematic demonstration of the main message of our work.

To investigate the mechanism by which hUC-MSC-derived CHI3L1 regulates T cell immunity and alleviates Con A-induced liver injury, we performed RNA sequencing analysis of activated T cells cultured alone (T cells) or in the presence of hUC-MSCs with or without CHI3L1 knockdown (T + MSC^shNTC^ or T + MSC^sh*CHI3L1*^), and conducted gene set enrichment analysis (GSEA). The IL6-JAK-STAT3 signalling pathway was one of the most significantly enriched pathways in the T + MSC^sh*CHI3L1*^ group compared with the T + MSC^shNTC^ group (Fig. 6A and 6C). Interestingly, this pathway was repressed in T cells co-cultured with MSC^shNTC^ (T + MSC^shNTC^ group) compared to activated T cells (Fig. 6B and 6D). Previous studies have shown that activation of the JAK/STAT system is important for T cell activation, proliferation, and function^23^. Thus, we hypothesised that CHI3L1 expressed in hUC-MSCs suppressed T cell function by inhibiting the JAK/STAT signalling pathway in T cells (Fig. 7).

To validate this hypothesis, we performed western blot analysis for phosphorylated STAT1 and STAT3, which are hallmarks of JAK/STAT signalling pathway activation, in activated human T cells cultured alone or in the presence of hUC-MSCs with or without CHI3L1 knockdown. Consistent with the RNA sequencing data, phosphorylated STAT1/3 was upregulated upon T cell activation, while it was significantly inhibited in T cells co-cultured with MSC^shNTC^ and was recovered in the hUC-MSC^sh*CHI3L1*^ co-cultured group (Fig. 6E). In contrast, rCHI3L1 decreased STAT1/3 phosphorylation in activated T cells (Fig. 6F). These results showed that hUC-MSCs expressed CHI3L1 inhibited T cell function by inhibiting the JAK/STAT pathway.

Previous studies showed that CHI3L1 suppresses inflammation and ER stress by inducing PPARδ^24^, and PPARδ could inhibit the IL6-induced phosphorylation of STAT3 both in vitro and in vivo^25–27^. We examined our RNA sequencing data and found that PPARδ was upregulated in T cells co-cultured with MSC^shNTC^ compared to activated T cells but was decreased in T cells co-cultured with MSC^sh*CHI3L1*^ (Fig. 6G), which is in contrast to the phosphorylated STAT1/3 levels. Thus, we measured the expression of PPARδ in T cells cultured alone and in the presence of MSC^shNTC^ or MSC^sh*CHI3L1*^. Both RT-qPCR and western blot showed that MSC^shNTC^ could increase the expression of PPARδ in T cells, while this effect was mitigated upon CHI3L1 knockdown in hUC-MSCs (Fig. 6H-J).

We also measured phosphorylated STAT1/3 and PPARδ expression in liver MNCs in Con A-induced mouse models. Consistent with in vitro cultured T cells, phosphorylation of STAT1/3 was decreased in MNCs in the MSC^shNTC^ group, and recovered to levels comparable to those of PBS group in the MSC^sh*CHI3L1*^ treatment group, while PPARδ showed an opposite trend (Fig. 6K). These results indicated that hUC-MSCs secreted CHI3L1 may inhibit the JAK/STAT signalling pathway by inducing PPARδ. Next, we performed western blot for phosphorylated STAT1/3 in naïve T cells, activated T cells (stimulated with PHA), activated T cells co-cultured with hUC-MSCs, and activated T cells co-cultured with hUC-MSCs in the presence of PPARδ inhibitor (GSK3787) (Fig. 6L). The results showed that the inhibition of STAT1/3 phosphorylation by hUC-MSCs was abrogated by PPARδ inhibitor (GSK3787). Then, we used a PPARδ agonist (GW501516) to treat T cells in vitro. As expected, GW501516 inhibited STAT1 and STAT3 phosphorylation and inflammatory cytokine (IFN-γ and TNF-α) secretion in T cells (Fig. 6M-O), supporting the conclusion that PPARδ is the mediator of STAT pathway inhibition by hUC-MSCs. The above results showed that hUC-MSCs alleviated Con A-induced hepatitis by modulating T cells through CHI3L1/PPARδ/STAT1/3 signalling (Fig. 7).

## Discussion

The beneficial effects of MSC transplantation in various liver diseases, including liver fibrosis/cirrhosis, chronic-on-acute liver failure, fulminant hepatitis, ischemia-reperfusion injury and other complications after liver transplantation and AIH, have been reported in both animal models and clinical studies^5,28,29^. The therapeutic effects of MSCs are considered to exert through either direct cell-to-cell contact or paracrine secretion of soluble factors^30,31^. However, the underlying mechanisms by which MSCs improve liver diseases were still not yet been fully elucidated. In this study, we identified CHI3L1 as a novel mediator of the immunosuppressive effects of hUC-MSCs and the therapeutic effect of hUC-MSCs on Con A-induced liver injury in mice. CHI3L1 could inhibit T cell activation, proliferation, and inflammatory cytokine secretion, which contributed to alleviation of immune-mediated liver injury by MSCs. More importantly, rCHI3L1 could also suppress T cell responses and improve Con A-induced liver injury. These results suggested that CHI3L1 acted as a novel immunosuppressive mediator in immune-mediated liver diseases, and might serve as a target for developing therapeutic strategies to treat immune-mediated liver injury, even other T cell-mediated diseases.

CHI3L1 is considered to be an important pro-inflammatory mediator, as it is often elevated in both the serum and tissues of patients with inflammatory conditions^12,32^, including a range of liver diseases^33^. CHI3L1 is highly expressed in liver tissues and is induced in both acute and chronic liver injury in animal models^34^. Studies using knockout mice showed that deletion of CHI3L1 protects against ethanol-induced liver injury^35^ and liver fibrosis^36^. However, other groups reported that CHI3L1 protected livers from APAP-induced liver injury by inhibiting the secretion of inflammatory factors and macrophage infiltration^37^. These results indicated that the role of CHI3L1 in liver diseases may vary depending on the aetiology and pathogenesis of liver injuries, and it would be interesting to delineate the detailed roles of CHI3L1 in different liver injuries using cell type-specific knockout models.

During the Con A-induced liver injury, CHI3L1 increased significantly and induced the tissue factor that promoted intrahepatic activation of coagulation and tissue injury. However, deletion of CHI3L1 increased the serum levels of TNF-α after Con A treatment^38^, indicating that CHI3L1 might play multiple roles in this model. Emerging evidence indicates that T cell-produced pro-inflammatory cytokines, including TNF-α and IFN-γ, are mediators of inflammatory deregulation in various liver diseases^18,39^. Combined with the fact that CHI3L1 negatively regulates T cell activation^40^, we speculate that CHI3L1 might serve as a negative regulator of T cell response in Con A-induced liver injury. Our results showed that MSC-derived CHI3L1 or rCHI3L1, administrated followed the Con A administration, could significantly supressed T cell activation and pro-inflammatory cytokine production to mitigate the immune-mediated liver injury. Taken together, CHI3L1 acted as a negative regulator of immune response in Con A-induced liver injury.

Indeed, CHI3L1 functions as an anti-inflammatory factor in most cases, which is consistent with our findings that CHI3L1 is a mediator of the immunosuppression functions of hUC-MSCs. CHI3L1 plays a negative role in T cell activation, especially Th1 cytokine expression^40^, inhibits macrophage pyroptosis and inflammasome activation^6^. In addition, knockout of CHI3L1 in donor cells dramatically exacerbated acute graft-versus-host disease by promoting Tfh differentiation and Tfh-related cytokine secretion^41^. More recently, it was reported that cancer-associated fibroblast-derived CHI3L1 enhances the migration and growth of breast cancer by suppressing the T cell population and IFN-γ and TNF-α expression^10^. Another interesting finding is that, consistent with the previous study showing that CHI3L1 activates the TGFβ pathway in different contexts^42,43^, we also observed a positive correlation of the TGFβ pathway in T cells co-cultured with MSC^shNTC^ compared to T cells cultured with MSC^sh*CHI3L1*^ (data not shown). As TGFβ is a canonical pathway for inducing regulatory T (Treg) cells, it would be interesting to investigate whether CHI3L1 mediates the immunosuppressive effects of hUC-MSCs by inducing Treg cells in the future.

The function of CHI3L1 is mediated by two putative cell surface receptors, CRTH2 and IL-13Rα2^44^. However, the intracellular downstream effector of CHI3L1 remains to be characterised. Our RNA sequencing data showed that STAT signalling (including IL2/STAT5 and JAK/STAT3) were the top signalling pathways affected in T cells co-cultured with CHI3L1-knockdown hUC-MSCs. Interestingly, consistent with our observation, previous studies showed that STAT1/5 phosphorylation was increased in CHI3L1-knockout T cells^40^. STAT signalling pathways were reported to be important for the proliferation, differentiation, survival, and apoptosis of various cells, including T lymphocytes^45^. Hence, we hypothesised that the STAT signalling pathway might contribute to the immunosuppressive effects of CHI3L1 on T cells. This hypothesis was further validated in vitro and in vivo. A positive correlation between PPARδ expression and STAT3 phosphorylation has been noted previously^46^. CHI3L1 was reported to ameliorate LPS-induced atherosclerotic reactions via PPARδ-mediated suppression of inflammation and ER stress^24^. Therefore, we hypothesised that the suppression of STAT phosphorylation might be the consequence of CHI3L1-induced PPARδ expression. Previous studies showed that CHI3L1 induced Wnt/β-catenin, Erk1/2, Akt signaling and STAT6 activation^6,11,43,47–49^, and all of these pathways were evidenced to be able to induce PPARδ expression in different cell contexts^50–53^. We found that MSC-derived CHI3L1 indeed promoted PPARδ expression and decreased the phosphorylation of STAT1/3 in T cells and the PPARδ agonist could inhibit the function of T cells. In contrast, PPARδ inhibitor abrogated the inhibition of STAT1/3 phosphorylation in T cells by hUC-MSCs. These findings demonstrated that the PPARδ/STAT1/3 axis is a target of CHI3L1 and elucidated the intracellular downstream effectors of CHI3L1.

## Materials and methods

### Isolation and expansion of hUC-MSCs

Umbilical cord collection and processing were approved by the Institutional Human Ethics Committee of The Third Affiliated Hospital of Sun Yat-sen University. Informed consent was obtained from all participants included in the study. MSCs were isolated and expanded from human umbilical cord according to the protocol reported previously by Bhonde RR et al*.*^54^. In brief, the UC was dissected and the umbilical cord vein and arteries were manually removed. The UC tissues and Wharton’s jelly were minced into small fragments of about 2 mm diameter and were cultured in L-DMEM (Gibco) supplemented with 10% FBS, 1% glutamine, 1% penicillin/streptomycin and kept at 37 °C with 5% CO2 in a water-jacketed incubator. The hUC-MSCs started to migrate from the explants within 7–12 days. Cells were cultured for 3 more days and non-adherent cells were removed by changing the medium completely. The rest of the adherent cells were hUC-MSCs.

### RNA isolation and quantitative real-time PCR

Total RNA was extracted with TRIzol reagent (Invitrogen, Carlsbad, CA), followed by reverse transcription (RT) with the RevertAid First Strand cDNA Synthesis Kit (Thermo Scientific). cDNA was used as the template in real-time PCR with SYBR Green reagent (Catalog #4887352001, SYBR Green I Master, Roche) to determine specific gene expression. All reactions were performed in triplicates and GAPDH was used as the internal control. The relative mRNA abundance was calculated using the ΔΔCt methods. Primers were listed in Supplementary Table 1.

### Western blot

Whole-cell lysate of MSCs in 6-well plates or CD3^+^ T cells in 24-well plates were prepared using lysis buffer containing 62.5 mM Tris-HCl (PH = 6.8), 2% SDS, 10% glycerol, 0.02% bromophenol blue and 50 mM DTT. Proteins were separated by 8% or 10% sulfate-polyacrylamide gel electrophoresis. After the separation, proteins were transferred to polyvinylidene fluoride membrane, blocked with TBST containing 5% non-fat dry milk, and analysed for target proteins with specific primary antibodies. Antibodies were listed in Supplementary Table 2.

### RNA sequencing

The sample preparation, sequencing, and data analysis were performed as previously described^55^ and the sequencing data are available in the Gene Expression Omnibus database under the accession number GSE166327.

### Gene knockdown using shRNA

Short hairpin RNAs (shRNAs) were designed, synthesised (Sangon Biotech, Shanghai, China), and cloned into pLKO.1_TRC Cloning Vector (addgene #8453). MSCs were transduced with lentiviral supernatants generated from HEK293T cells with the addition of 8 μg/ml polybrene (TR-1003, Sigma-Aldrich). Non-targeting sequence construct was used as the negative control. shRNA sequences were listed in Supplementary Table 3.

### Flow cytometry

Flow cytometric analysis was performed on LSR II (BD) or CytoFLEX flow cytometer (Beckman Coulter, Fullerton, CA, USA), and data were analysed with FlowJo7.6 software (Treestar, Ashland, OR, USA) or CytoExpert software (Beckman Coulter). Anti-human CD73-FITC (Catalog #561254), CD105-FITC(Catalog #561443), CD44-APC (Catalog #559942), CD34-PE-Cy7 (Catalog #560710), CD45-PE-Cy7 (Catalog #557748), and corresponding isotype control antibodies were purchased from BD Bioscience. Anti-human CD3-FITC (Catalog #555332), CD8-APC (Catalog #555369), CD4-BV421 (Catalog #562424), anti-human TNF-α-PE (Catalog #502909), anti-human IFN-γ-PE-Cy7 (Catalog #557643) were purchased from BD Pharmingen. Fixation/Permeabilization Solution Kit (Catalog #554714, BD Pharmingen) was used for staining intracellular cytokines.

### Differentiation assays

For osteogenic and adipogenic differentiation, MSCs were seeded in L-DMEM complete medium before induction. When the cells reached 80–90% confluence, the medium was replaced with bone induction medium containing L-DMEM, 10% (v/v) foetal calf serum, 2 mM glutamine, 100 IU/mL penicillin, 100 mg/mL streptomycin, dexamethasone (0.1 μM, Merck), ascorbic acid (50 μg/mL, Sigma-Aldrich) and β-glycerol phosphate (10 mM, Sigma-Aldrich). After 2–3 weeks, osteogenic differentiation was identified by mineralisation of extracellular matrix and calcium deposits with 0.5% Alizarin Red S (v/v) (Sigma-Aldrich) staining. Adipogenic differentiation was induced with adipogenic induction medium containing H-DMEM (Gibco), 10% (v/v) foetal calf serum, 2 mM glutamine, 100 IU/mL penicillin, 100 mg/mL streptomycin, dexamethasone (1 μM, Merck), insulin (10 μg/mL, Prospect), isobuthylmethylxanthine (IBMX, 0.5 mM, Sigma-Aldrich) and indomethacin (0.2 mM, Sigma-Aldrich) when the cells reached 100% confluence. Adipogenic differentiation was verified by the typical production of lipid droplets staining with Oil Red O (O0625, Sigma-Aldrich).

### Lymphocytes proliferation assays

MSCs (1 × 10^5^ cells) were plated into 24-well plate (Corning) and cultured 24 h before co-culture with lymphocytes. The next day, human peripheral blood mononuclear cells (MNCs) were collected from healthy donors and red blood cells were removed by ammonium chloride lysis buffer for 5 min at room temperature, and then centrifuged at 300 g for 5 min. After washed twice with PBS (containing 3% fetal calf serum (FCS)), cells were incubated with an anti-mouse CD3 antibody (Catalog #100204, Biolegend) at 4 °C for 30 min. CD3^+^ T cells were sorted by CD3 MicroBeads (Catalog #130-050-101, Miltenyi Biotec, Germany). 5, 6-carboxyfluorescein diacetate succinimidyl ester (CFSE, Invitrogen, Carlsbad, CA) staining was used to assess CD3^+^ T cell proliferation according to the manufacture’s instruction. Briefly, CD3^+^ T cells were re-suspended at 5 × 10^6^ cells/mL in PBS containing 0.1% BSA, then CFSE was added at a final concentration of 5 μM and the cell suspension was incubated in dark for 10 min in the water bath at 37 °C. Labelling was stopped by washing with cold RPMI1640 (Hyclone) containing 10% (v/v) FCS. CD3^+^ T cells were suspended in RPMI1640 at 2.5 × 10^6^ cells/mL and add 1 mL to the 24-well plates in the presence or absence of MSCs. T cell proliferation was induced by phytohaemagglutinin (PHA, Sigma-Aldrich) at the final concentration of 5 μg/mL. After co-culture for 3 days, CD3^+^ T cells were collected and analysed by flow cytometry (CytoFLEX, Beckman Coulter). To investigate the inhibition of MSCs on the CD3^+^ T cell activation, the collected cells were also stained with anti-mouse CD3 (Catalog #100336, Biolegend), CD69 (Catalog #104506, Biolegend) and CD25 antibodies (Catalog #102016, Biolegend).

### Intracellular cytokine assays

For in vitro experiments, purified CD3^+^ T cells were cultured in the presence or absence of MSCs at ratios of 5:1. TNF-α and IFN-γ were analysed by flow cytometer after 2 days. 6 h before analysis, cells were stimulated with PMA (50 ng/mL) plus ionomycin (500 ng/mL), and brefeldin A (BFA, 10 μg/mL) was used to inhibit the secretion of cytokines. PMA, ionomycin, and BFA all obtained from Sigma-Aldrich. For in vivo experiments, liver MNCs were isolated 24 h after Con-A administration and directly analysed for TNF-α (506306, Biolegend) and IFN-γ (554412, BD Pharmingen) with flow cytometer.

### Animals

C57BL/6J mice were housed under specific pathogen-free conditions. Experiments were performed with female animals at 6–8 weeks of age under ethical conditions approved by the Institutional Animal Care and Use Committee of The Third Affiliated Hospital of Sun Yat-sen University. Sample size was calculated based on the resource equation approach, which is widely used to determine the sample size in animal studies by calculating the minimum and maximum numbers of animals required.

### Con A-induced hepatitis and cell transplantation

A single dose of Con A (Sigma-Aldrich) was administrated at 15 or 20 mg/kg through the tail vein. PBS or 1 × 10^6^ hUC-MSCs (MSC^shNTC^ or MSC^sh*CHI3L1*^ (*N* = 10)) suspended in 200 μL PBS were transplanted intravenously (i.v) 30 min after Con A injection. Blood and liver tissues were collected 24 h later for further analysis. For recombinant CHI3L1 study, PBS or rCHI3L1 (100 ng/mL) was administrated and observed.

### Hematoxylin and eosin (H&E) staining

Liver tissues were fixed in 4% paraformaldehyde and embedded in paraffin. 4 µm sections were prepared and stained with H&E.

### Serum alanine aminotransferase (ALT) and aspartate transaminase (AST) measurement

Mouse serum samples were obtained at 24 h post Con A injection. Serum ALT and AST was measured using Hitachi 7020 automatic biochemical analyzer (Hitachi, Tokyo, Japan).

### Analysis of serum cytokines

IFN-γ, TNF-α, and IL6 concentrations in serum were assessed using Cytometric Bead Array (CBA) kits (BD biosciences, USA) according to the manufacturer’s instructions.

### Isolation of liver MNCs

Liver tissues were minced to dissociate cells followed by filtration through a 200 µm pore mesh. Hepatocytes were removed by centrifuging at 50 *g* for 2 min and the supernatant was collected. After centrifuging at 580 *g* for 10 min, the cell pellet was re-suspended in the culture medium. For the isolation of MNCs, the cell pellet was re-suspended with a 40% Percoll solution and overlaid on a 70% Percoll solution. After centrifuging at 2000 *rpm* for 30 min, the interphase was collected and re-suspended in the culture medium.

### Statistical analysis

No statistical methods were used to predetermine the sample sizes. No data exclusions were made in experimental sections. No data show significant deviation from normal distribution and data from different treatment groups show good homogeneity of variances. The exact sample size for each experimental group has been shown in figure legends. All results were expressed as mean ± SD. Statistical comparison was made by two-tailed Student’s *t* test between two groups or one-way ANOVA for multi-group comparison. Survival was analysed by the Kaplan–Meier log-rank test. *P* < 0.05 was considered significant. Analyses and graphs were performed using GraphPad Prism version 7 (San Diego, CA).

## Supplementary information

Supplementary Figure 1

supplemententary figure legend

Table S1

Supplementary Table 2

Table S3

## References

[1] Eksteen B, Afford SC, Wigmore SJ, Holt AP, Adams DH (2007). Immune-mediated liver injury. Semin. Liver Dis..

[2] Wang M, Yuan Q, Xie L (2018). Mesenchymal Stem Cell-Based Immunomodulation: Properties and Clinical Application. Stem Cells Int..

[3] Hu C, Zhao L, Li L (2019). Current understanding of adipose-derived mesenchymal stem cell-based therapies in liver diseases. Stem Cell Res Ther..

[4] Kholodenko IV, Kurbatov LK, Kholodenko RV, Manukyan GV, Yarygin KN (2019). Mesenchymal Stem Cells in the Adult Human Liver: Hype or Hope?. Cells.

[5] de Miguel MP, Prieto I, Moratilla A, Arias J, Aller MA (2019). Mesenchymal Stem Cells for Liver Regeneration in Liver Failure: From Experimental Models to Clinical Trials. Stem Cells Int.

[6] He CH (2013). Chitinase 3-like 1 regulates cellular and tissue responses via IL-13 receptor alpha2. Cell Rep..

[7] Capone M (2016). Chitinase 3-like-1 is produced by human Th17 cells and correlates with the level of inflammation in juvenile idiopathic arthritis patients. Clin. Mol. Allergy.

[8] Low D (2015). Chitinase 3-like 1 induces survival and proliferation of intestinal epithelial cells during chronic inflammation and colitis-associated cancer by regulating S100A9. Oncotarget.

[9] Huang H (2015). CHI3L1 Is a Liver-Enriched, Noninvasive Biomarker That Can Be Used to Stage and Diagnose Substantial Hepatic Fibrosis. OMICS.

[10] Cohen N (2017). Fibroblasts drive an immunosuppressive and growth-promoting microenvironment in breast cancer via secretion of Chitinase 3-like 1. Oncogene.

[11] Im JH (2020). Deletion of Chitinase-3-like 1 accelerates stroke development through enhancement of Neuroinflammation by STAT6-dependent M2 microglial inactivation in Chitinase-3-like 1 knockout mice. Exp. Neurol..

[12] Kim DH, Choi JM (2018). Chitinase 3-like-1, a novel regulator of Th1/CTL responses, as a therapeutic target for increasing anti-tumor immunity. BMB Rep..

[13] Breyne K (2018). Immunomodulation of Host Chitinase 3-Like 1 During a Mammary Pathogenic Escherichia coli Infection. Front. Immunol..

[14] Lieder R, Sigurjonsson OE (2014). The Effect of Recombinant Human Interleukin-6 on Osteogenic Differentiation and YKL-40 Expression in Human, Bone Marrow-Derived Mesenchymal Stem Cells. Biores Open Access.

[15] Hoover DJ (2013). Expression of the chitinase family glycoprotein YKL-40 in undifferentiated, differentiated and trans-differentiated mesenchymal stem cells. PLoS ONE.

[16] da Silva Meirelles L, Chagastelles PC, Nardi NB (2006). Mesenchymal stem cells reside in virtually all post-natal organs and tissues. J. Cell Sci..

[17] Deans RJ, Moseley AB (2000). Mesenchymal stem cells: biology and potential clinical uses. Exp. Hematol..

[18] Kato J (2013). Interferon-gamma-mediated tissue factor expression contributes to T-cell-mediated hepatitis through induction of hypercoagulation in mice. Hepatology.

[19] Tiegs G, Hentschel J, Wendel AA (1992). T cell-dependent experimental liver injury in mice inducible by concanavalin A. J. Clin. Investig..

[20] Wang W (2018). Interleukin-35 Gene-Modified Mesenchymal Stem Cells Protect Concanavalin A-Induced Fulminant Hepatitis by Decreasing the Interferon Gamma Level. Hum. Gene Ther..

[21] Han X (2014). Interleukin-17 enhances immunosuppression by mesenchymal stem cells. Cell Death Differ..

[22] Zheng C, Yin S, Yang Y, Yu Y, Xie X (2018). CD24 aggravates acute liver injury in autoimmune hepatitis by promoting IFN-gamma production by CD4(+) T cells. Cell Mol. Immunol..

[23] Waldmann TA, Chen J (2017). Disorders of the JAK/STAT Pathway in T Cell Lymphoma Pathogenesis: Implications for Immunotherapy. Annu Rev. Immunol..

[24] Jung TW (2018). Chitinase-3-like protein 1 ameliorates atherosclerotic responses via PPARdelta-mediated suppression of inflammation and ER stress. J. Cell Biochem.

[25] Lo SH (2017). Ginsenoside Rh2 Improves Cardiac Fibrosis via PPARdelta-STAT3 Signaling in Type 1-Like Diabetic Rats. Int. J. Mol. Sci.

[26] Serrano-Marco L (2012). The peroxisome proliferator-activated receptor (PPAR) beta/delta agonist GW501516 inhibits IL-6-induced signal transducer and activator of transcription 3 (STAT3) activation and insulin resistance in human liver cells. Diabetologia.

[27] Serrano-Marco L (2011). Activation of peroxisome proliferator-activated receptor-beta/-delta (PPAR-beta/-delta) ameliorates insulin signaling and reduces SOCS3 levels by inhibiting STAT3 in interleukin-6-stimulated adipocytes. Diabetes.

[28] El Agha E (2017). Mesenchymal Stem Cells in Fibrotic Disease. Cell Stem Cell.

[29] Alfaifi M, Eom YW, Newsome PN, Baik SK (2018). Mesenchymal stromal cell therapy for liver diseases. J. Hepatol..

[30] Aggarwal S, Pittenger MF (2005). Human mesenchymal stem cells modulate allogeneic immune cell responses. Blood.

[31] Shi Y (2018). Immunoregulatory mechanisms of mesenchymal stem and stromal cells in inflammatory diseases. Nat. Rev. Nephrol..

[32] Jiang Z (2020). The clinical significance of serum chitinase 3-like 1 in hepatitis B-related chronic liver diseases. J. Clin. Lab Anal..

[33] Kjaergaard AD, Johansen JS, Bojesen SE, Nordestgaard BG (2016). Role of inflammatory marker YKL-40 in the diagnosis, prognosis and cause of cardiovascular and liver diseases. Crit. Rev. Clin. Lab Sci..

[34] Pizano-Martinez O (2011). YKL-40 expression in CD14(+) liver cells in acute and chronic injury. World J. Gastroenterol..

[35] Lee DH (2019). Chitinase-3-like-1 deficiency attenuates ethanol-induced liver injury by inhibition of sterol regulatory element binding protein 1-dependent triglyceride synthesis. Metabolism.

[36] Higashiyama M (2019). Chitinase 3-like 1 deficiency ameliorates liver fibrosis by promoting hepatic macrophage apoptosis. Hepatol. Res..

[37] Wang Y, Zhong M, Wang W, Li YH (2019). Chi3l1 regulates APAP-induced liver injury by promoting macrophage infiltration. Eur. Rev. Med. Pharm. Sci..

[38] Shan Z (2018). Chitinase 3-like-1 promotes intrahepatic activation of coagulation through induction of tissue factor in mice. Hepatology.

[39] Gantner F, Leist M, Lohse AW, Germann PG, Tiegs G (1995). Concanavalin A-induced T-cell-mediated hepatic injury in mice: the role of tumor necrosis factor. Hepatology.

[40] Kim DH (2018). Regulation of chitinase-3-like-1 in T cell elicits Th1 and cytotoxic responses to inhibit lung metastasis. Nat. Commun..

[41] Li Z (2017). Chitinase 3-Like-1-Deficient Splenocytes Deteriorated the Pathogenesis of Acute Graft-Versus-Host Disease via Regulating Differentiation of Tfh Cells. Inflammation.

[42] Qiu QC (2018). CHI3L1 promotes tumor progression by activating TGF-beta signaling pathway in hepatocellular carcinoma. Sci. Rep..

[43] Lee CM (2016). IL-13Ralpha2 uses TMEM219 in chitinase 3-like-1-induced signalling and effector responses. Nat. Commun..

[44] Zhou Y (2015). Chitinase 3-like-1 and its receptors in Hermansky-Pudlak syndrome-associated lung disease. J. Clin. Investig..

[45] Jaruga B, Hong F, Kim WH, Gao B (2004). IFN-gamma/STAT1 acts as a proinflammatory signal in T cell-mediated hepatitis via induction of multiple chemokines and adhesion molecules: a critical role of IRF-1. Am. J. Physiol. Gastrointest. Liver Physiol..

[46] Sun L (2018). PPAR-delta modulates membrane cholesterol and cytokine signaling in malignant B cells. Leukemia.

[47] Zhou Y (2018). Galectin-3 Interacts with the CHI3L1 Axis and Contributes to Hermansky-Pudlak Syndrome Lung Disease. J. Immunol..

[48] Kim EG (2020). Chitinase 3-Like 1 Contributes to Food Allergy via M2 Macrophage Polarization. Allergy Asthma Immunol. Res..

[49] Geng B (2018). Chitinase 3-like 1-CD44 interaction promotes metastasis and epithelial-to-mesenchymal transition through beta-catenin/Erk/Akt signaling in gastric cancer. J. Exp. Clin. Cancer Res..

[50] Xie H (2008). Inactivation of nuclear Wnt-beta-catenin signaling limits blastocyst competency for implantation. Development.

[51] Yano M (2007). Statins activate peroxisome proliferator-activated receptor gamma through extracellular signal-regulated kinase 1/2 and p38 mitogen-activated protein kinase-dependent cyclooxygenase-2 expression in macrophages. Circ. Res.

[52] Wang D (2004). Prostaglandin E(2) promotes colorectal adenoma growth via transactivation of the nuclear peroxisome proliferator-activated receptor delta. Cancer Cell.

[53] Geng T (2020). CD137 signaling induces macrophage M2 polarization in atherosclerosis through STAT6/PPARdelta pathway. Cell Signal.

[54] Chandravanshi B, Bhonde RR (2018). Human Umbilical Cord-Derived Stem Cells: Isolation, Characterization, Differentiation, and Application in Treating Diabetes. Crit. Rev. Biomed. Eng..

[55] Chen X (2019). Human Mesenchymal Stem Cell-Treated Regulatory CD23(+)CD43(+) B Cells Alleviate Intestinal Inflammation. Theranostics.

