# Supplementary figures and images for: Mesenchymal stem cells alleviate experimental immune-mediated liver injury via chitinase 3-like protein 1-mediated T cell suppression
Source: Cell Death Dis. 2021 Mar 4;12(3):240. doi: 10.1038/s41419-021-03524-y (PMC7933182; doi:10.1038/s41419-021-03524-y)

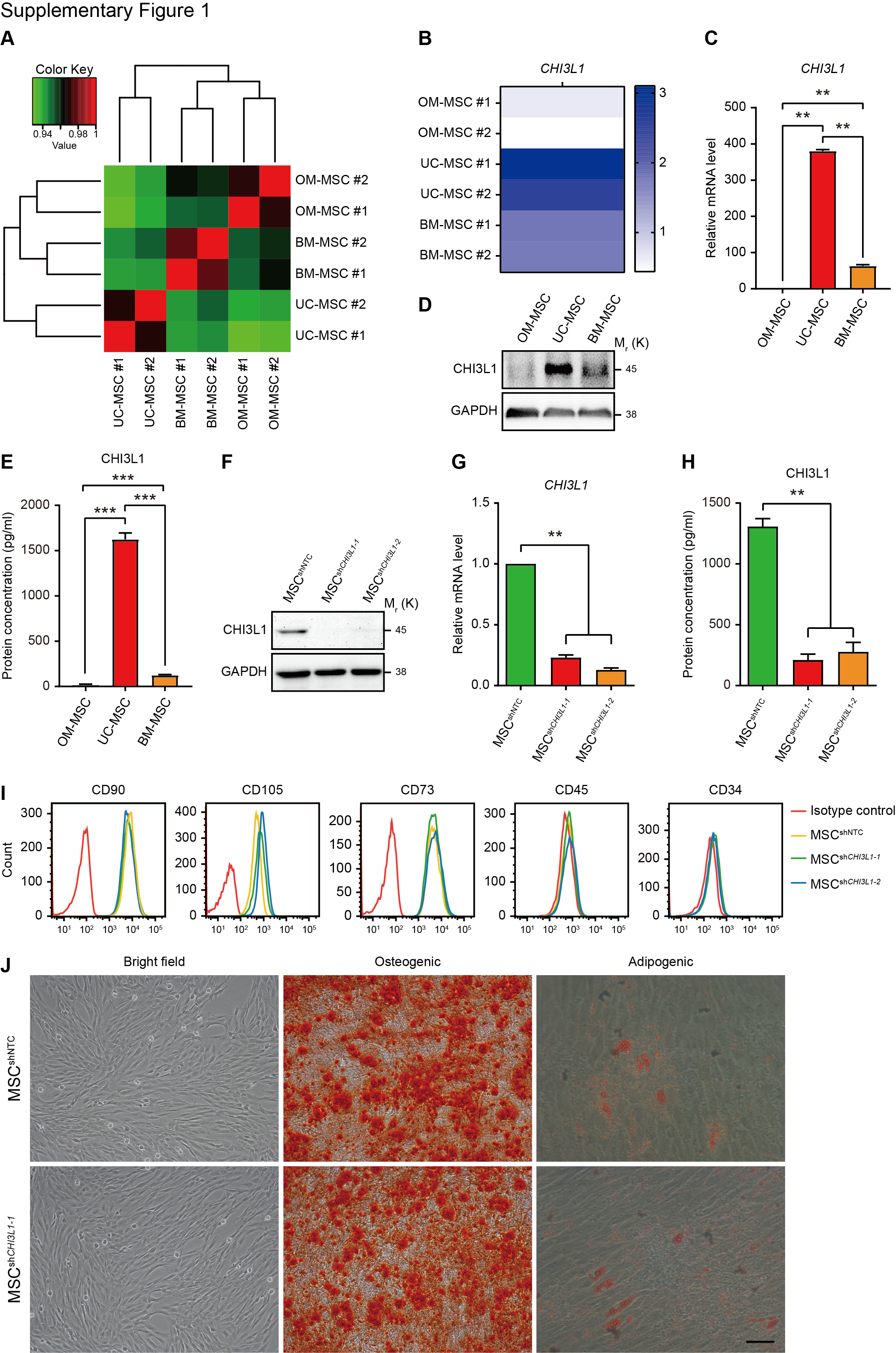

Supplement: Supplementary file 1 — Supplementary Figure 1 [file 41419_2021_3524_MOESM1_ESM.png]
