# Supplementary material for: Mesenchymal stem cells alleviate experimental immune-mediated liver injury via chitinase 3-like protein 1-mediated T cell suppression
Source: Cell Death Dis. 2021 Mar 4;12(3):240. doi: 10.1038/s41419-021-03524-y (PMC7933182; doi:10.1038/s41419-021-03524-y)
Supplement: Supplementary file 2 — supplemententary figure legend [file 41419_2021_3524_MOESM2_ESM.docx]

**Supplementary Figure legends**

**Supplementary Figure 1. CHI3L1 is highly expressed in hUC-MSCs and down-regulating CHI3L1 does not change the characteristics of hUC-MSCs.** (A) Heatmap showing sample correlation of RNA-sequencing data for human OM-MSCs, UC-MSCs, and BM-MSCs. (B) Heatmap of RNA-sequencing data showing the expression of *CHI3L1* in human OM-MSCs, UC-MSCs, and BM-MSCs. (C) qPCR for *CHI3L1* in human OM-MSCs, UC-MSCs, and BM-MSCs, the values were referred to OM-MSCs. (D) Western blot for detecting CHI3L1 expression in human OM-MSCs, UC-MSCs, and BM-MSCs. (E) ELISA for testing the levels of CHI3L1 in supernatants of hUC-MSCs, BM-MSCs and OM-MSCs. (F) Western blot for testing CHI3L1 knockdown efficiency in hUC-MSCs transduced with shNTC or 2 shRNAs for *CHI3L1*. ShNTC was used as a control (also hereafter in similar experiments). GAPDH was used as a loading control (also hereafter in similar experiments). (G) qPCR for testing *CHI3L1* knockdown efficiency in hUC-MSCs transduced with shRNA for non-targeting sequence (shNTC) or 2 shRNAs for *CHI3L1*. (H) ELISA for testing the levels of CHI3L1 in supernatants of hUC-MSCs transduced with shNTC or 2 shRNAs for *CHI3L1*. (I) Representative flow cytometry analysis for cell surface markers of hUC-MSCs transduced with shNTC or 2 shRNAs for *CHI3L1*. (J) Representative photographs showing adipogenic and osteogenic differentiation of hUC-MSCs transduced with shNTC or shRNA for *CHI3L1*. Scar bar = 100 μm. Data in (C), (E), (G), and (H) are shown as mean ± SD (n = 3 biological replicates) with the indicated significance (**p < 0.01).
