# Supplementary material for: Mesenchymal stem cells alleviate experimental immune-mediated liver injury via chitinase 3-like protein 1-mediated T cell suppression
Source: Cell Death Dis. 2021 Mar 4;12(3):240. doi: 10.1038/s41419-021-03524-y (PMC7933182; doi:10.1038/s41419-021-03524-y)
Supplement: Supplementary file 3 — Table S1 [file 41419_2021_3524_MOESM3_ESM.docx]

Table S1. Primers used for qPCR

| Genes Forward sequence(5'--3') | Reverse sequence(5'--3') |
| --- | --- |
| *GAPDH* GCACCGTCAAGGCTGAGAAC | TGGTGAAGACGCCAGTGGA |
| *CHI3L1* TCGCCGGACTTTCATCA | TCGGCCTTCATTTCCTTG |
| *PPARD* CTCTATCGTCAACAAGGACG | GTCTTCTTGATCCGCTGCAT |
