# Supplementary material for: Mesenchymal stem cells alleviate experimental immune-mediated liver injury via chitinase 3-like protein 1-mediated T cell suppression
Source: Cell Death Dis. 2021 Mar 4;12(3):240. doi: 10.1038/s41419-021-03524-y (PMC7933182; doi:10.1038/s41419-021-03524-y)
Supplement: Supplementary file 4 — Supplementary Table 2 [file 41419_2021_3524_MOESM4_ESM.docx]

Supplementary Table 2. Antibodies used for western blot

| CHI3L1 | R&D systems |
| --- | --- |
| p-STAT3(Tyr705) | Cell Signaling Technology |
| STAT3 (79D7) | Cell Signaling Technology |
| p-STAT1 (Ser727) | Cell Signaling Technology |
| STAT1 (D1K9Y) | Cell Signaling Technology |
| GAPDH | Cell Signaling Technology |
| PPARδ | Abclonal |
