# Supplementary material for: Mesenchymal stem cells alleviate experimental immune-mediated liver injury via chitinase 3-like protein 1-mediated T cell suppression
Source: Cell Death Dis. 2021 Mar 4;12(3):240. doi: 10.1038/s41419-021-03524-y (PMC7933182; doi:10.1038/s41419-021-03524-y)
Supplement: Supplementary file 5 — Table S3 [file 41419_2021_3524_MOESM5_ESM.docx]

Table S3. shRNA sequence used to generate lentiviral plasmids for RNA silencing

| ShNTC Oligonucleotide(5'--3')  Forward CCGGCGTACGCGGAATACTTCGACTCGAGTCGAAGTATTCCGCGTACGTTTTTG  Reverse AATTCAAAAAATCAAGTCAGTACCGCCATTT CTCGAG AAATGGCGGTACTGACTTGAT  Sh*CHI3L1*-1 Oligonucleotide(5'--3') |
| --- |
| Forward CCGGATCAAGTCAGTACCGCCATTTCTCGAGAAATGGCGGTACTGACTTGATTTTTTG  Reverse AATTCAAAAAATCAAGTCAGTACCGCCATTTCTCGAGAAATGGCGGTACTGACTTGAT |
| Sh*CHI3L1*-2 Oligonucleotide(5'--3') |
| Forward CCGGTAGCATCATGACCTACGATTTCTCGAGAAATCGTAGGTCATGATGCTATTTTTG |
| Reverse AATTCAAAAA TAGCATCATGACCTACGATTT CTCGAG AAATCGTAGGTCATGATGCTA |
